# Genetic role in autoimmune encephalitis and paraneoplastic neurological syndromes

**DOI:** 10.1097/WCO.0000000000001486

**Published:** 2026-04-17

**Authors:** Sophie N.M. Binks, Jeroan Yip, Julian C. Knight

**Affiliations:** a Nuffield Department of Clinical Neurosciences, University of Oxford; b Department of Neurology, John Radcliffe Hospital; c Department of Psychiatry, Warneford Hospital; d Centre for Human Genetics; e Chinese Academy of Medical Sciences Oxford Institute, University of Oxford, Oxford, UK

**Keywords:** autoimmune encephalitis, genome wide association study, human leucocyte antigen, leucine-rich glioma-inactivated 1, *N*-methyl-d-aspartate receptor

## Abstract

**Purpose of review:**

To synthesize evidence and recent advances concerning genetic contributors to autoimmune encephalitis (AE) and paraneoplastic neurological syndromes (PNS), relating these to disease processes and clinical management.

**Recent findings:**

The known immunogenetic role of the human leucocyte antigen (HLA) region – critical to immune and infection response – was refined. A multiethnic IgLON5 study revealed *DQ* associations, surpassing those previously demonstrated with *DR*, and arguing for T cell involvement. In PNS with anti-Hu antibodies, a DQB1*02:01~DRB1*03:01 haplotype was preferentially linked to a sensory neuropathy phenotype.

Outside the HLA, there were genome-wide association studies (GWAS) in the two commonest AEs. The first AE GWAS with discovery and validation cohorts took place in leucine-rich glioma-inactivated 1-antibody encephalitis (LGI1-Ab-E). This identified a risk locus in *PTPRD*, a protein tyrosine phosphatase with dual immune and brain activity, and a polygenic risk score (PRS). In *N*-methyl-d-aspartate receptor-antibody encephalitis (NMDAR-Ab-E), a discovery-only cohort implicated the type 1 interferon pathway gene *IFIH1.*

**Summary:**

Genetic status can perform as a biomarker or prioritize targets for drug development. The paucity of familial cases, the allele frequency of risk HLAs in healthy individuals, and the applicability of PRS, support a multihit model combining genetic and environmental risks.

## INTRODUCTION

In autoimmune encephalitis (AE) pathogenic autoantibodies are generated and directed against surface brain proteins [1]. These proteins play a critical role in normal central nervous system (CNS) pathophysiology and their blockade, at the molecular level, leads to loss of function or tissue damage [2]. Clinically, this manifests in devastating symptoms including seizures, personality change, and cognitive impairment [1,2]. While some AEs have established malignancy associations, most prominently *N*-methyl-d-aspartate receptor-antibody encephalitis (NMDAR-Ab-E) and ovarian teratoma in ~30% [3,4], they are classed as intermediate-risk paraneoplastic [5]. 

**Box 1:**
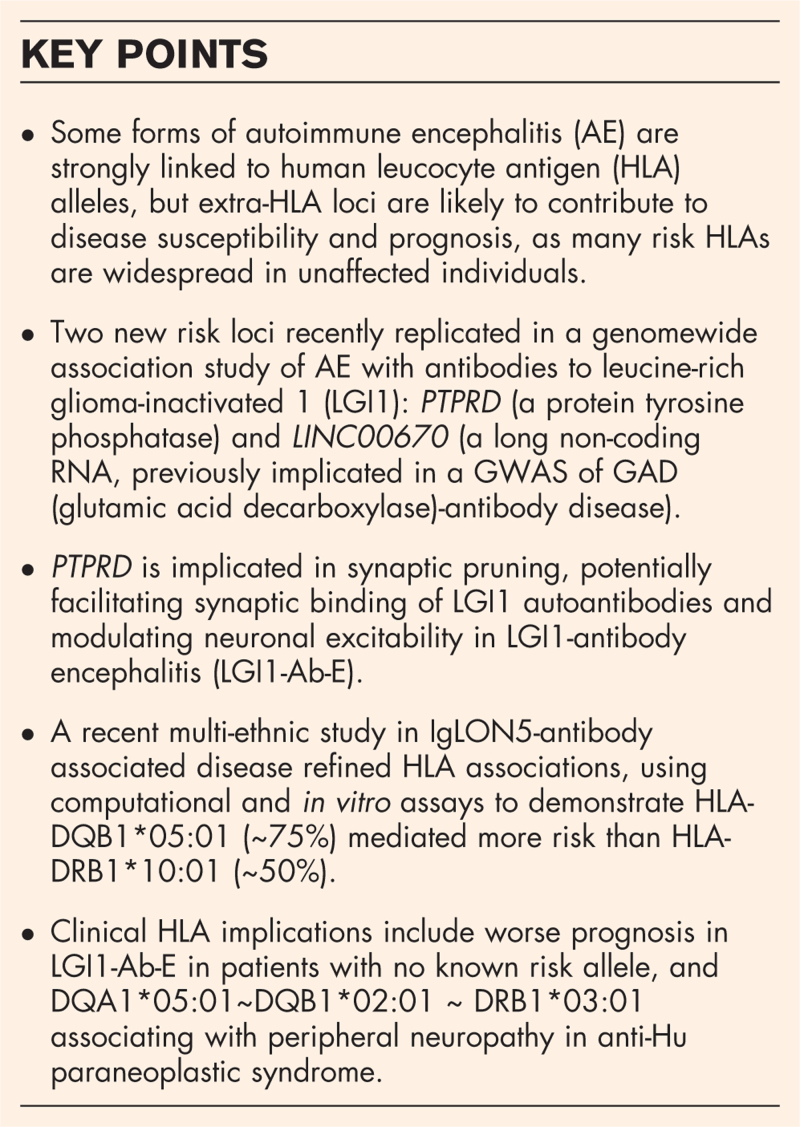
no caption available

Classical paraneoplastic neurologic syndromes (PNS) refers to a more heterogenous group of conditions, including limbic and rhombencephalitis, myelopathies, and neuro(no)pathies [5,6]. Autoantibodies in PNS are directed against intracellular entities; their direct pathogenic role is unconfirmed [5,6]. Clinical symptoms are usually attributed to T cell actions and the antibodies themselves may be an epiphenomenon, but they are tightly linked to and serve as critical biomarkers of specific cancer sub-types [5,6].

In keeping with this neuroimmune origin, to date most known AE and PNS genetic associations lie within the human leucocyte antigen (HLA) on chromosome 6 (Table 1). This is a critical region for infection and adaptive immunity, implicated in many autoimmune conditions [7]. Class 2 HLAs (DQ, DP and DR) are almost exclusive to professional antigen presenting cells (APCs), present exogenous 12–25 amino acid peptides to CD4^+^ T cells, and can invoke a B cell response. Most AE links are to HLA class 2, rather than class 1 (HLA-A, B and C), encoding antigen presenting molecules present on all nucleated cells and activating CD8^+^ cytotoxic T cells [7]. The strength of genetic association has led to some class 2 HLA alleles entering clinical practice as part of the diagnostic work-up [1]. A notable class 1 exception is the protective effect of the A*02 allele in the risk of postherpes simplex NMDAR-Ab-E (Table 1) [8,9].

**Table 1:** Human leucocyte antigen associations with autoimmune encephalitis and paraneoplastic syndromes

A | Autoimmune encephalitis with surface antibodies			
Antigen	HLA Association	Frequency	Notes
CASPR2	DRB1*11:01	Up to 90% in CASPR2-associated AE	Likely associated with central not peripheral disease
IgLON5	DQB1*05:01∼DRB1*10:01	∼75% DQB1*05:01, ∼50% DRB1*10:01	DQB1*05:01 is emerging as the causative allele and associated with CD4+ T cell activity
LGI1	DRB1*07:01 DRB1*04:02 DRB1*03:01	∼90% up to 10% ∼40%	Strong association across ethnicities In non DRB1*07:01 carriers, younger and female patients In a Chinese cohort of 34
NMDAR	DQB1*05:02, A*11:01, & A*02:07 DRB1*15:02, DRB1*16:02	15% for DQB1*05:02	In >400 Chinese patients; also recapitulated DRB1*16:02 Previously reported as weak associations in East Asian cohorts
Post-HSV1 NMDAR	A*02	∼20%	Protective HLA (frequency in healthy population ∼50%)

B | Autoimmune encephalitis with intracellular antibodies, including paraneoplastic syndromes			

Antigen	HLA Association	Frequency	Notes
AK5	DQA1*05:01∼DQB1*02:01∼DRB1*03:01	∼70%	
GAD65	DQA1*05:01∼DQB1*02:01∼DRB1*03:01	∼40%	An additional association with DQA1*03:01∼DQB1*03:02∼DRB1*04:01 was also described in a GWAS of 167 patients
Hu	DQA1*05:01∼DQB1*02:01∼DRB1*03:01 DRB4*01:01 **DQ2∼DR3**	∼40 across all cases ∼25% across all cases ∼50% across all cases	Enriched in peripheral disease/neuropathy Enriched in central disease Enriched in confirmed cancer
KLHL11	DQB1*02:01∼DRB1*03:01	60%	
Yo	DQA1*01:03∼DQB1*06:03∼DRB1*13: 01	∼30%	Only in patients with ovarian cancer

Bold font signifies new or significant updates in the last <2 years.

AE, autoimmune encephalitis; AK5, adenylate kinase 5; CASPR2, contactin-associated protein-like 2; CD4, cluster of differentiation 4; GAD65, glutamic acid decarboxylase 65; GWAS, genome-wide association study; HLA, human leukocyte antigen; HSV1, herpes simplex virus type 1; IgLON5, Immunoglobulin-like cell adhesion molecule 5; KLHL11, Kelch-like protein-11; LGI1, leucine-rich glioma-inactivated protein 1; NMDAR, N-methyl-D-aspartate receptor. Adapted and updated with permission, from Muniz-Castrillo and Honnorat (2024).

For example, around 90% of patients with leucine-rich glioma-inactivated 1 encephalitis (LGI1-Ab-E) carry HLA-DRB1*07:01, across a range of ancestries [10–12]. A separate Class 2 allele, HLA-DRB1*11:01, is found in patients with antibodies to contactin-associated protein-like 2 (CASPR2) [12]. The proportion may be as high as 90% in those with limbic encephalitis [13], while it is disputed whether it is enriched in other CASPR2 syndromes.

**FIGURE 1:**
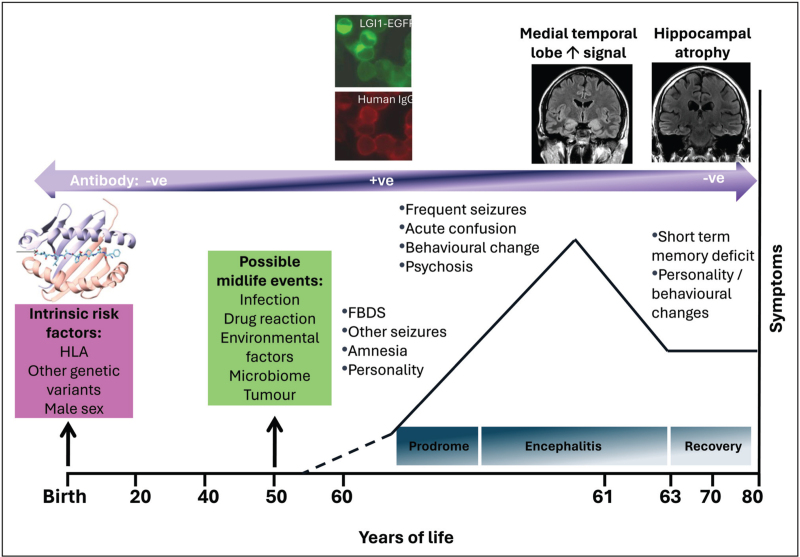
A multihit model of LGI1-antibody encephalitis. Schematic diagram to show the likely risk factors for developing LGI1 antibody encephalitis, possible midlife events, followed by the development of antibody positivity and onset of a prodrome of seizures, including FBDS and cognitive alterations. As these worsen into a full-blown encephalitic syndrome, medial temporal lobe hyperintensities are often noted. After immunotherapies, the disease often resolves to leave persistent cognitive deficits and hippocampal atrophy. EGFP, enhanced green fluorescent protein; FBDS, faciobrachial dystonic seizures; LGI1, leucine-rich glioma-inactivated 1. Reproduced with adaptation from Binks *et al.* [15]. This is an Open Access article distributed in accordance with the terms of the Creative Commons Attribution (CC BY 4.0) license, which permits others to distribute, remix, adapt and build upon this work, for commercial use, provided the original work is properly cited.

HLA-DRB1*07:01 homozygosity confers increased LGI1-Ab-E susceptibility [14], but more granular delineation of its potential role in disease trajectory has been pending. Moreover, these HLA alleles are common in healthy individuals, whereas AEs are rare, indicating that additional genetic and environmental contributors are required for disease initiation (Fig. 1) [12,15]. This review will highlight recent publications that have started to address these knowledge gaps, including genome-wide genetic associations beyond HLA alleles [16^▪▪^], which have also been reported in NMDAR-Ab-E [17^▪^].

During 2024–2026, other HLA associations, notably in patients with immunoglobulin-like cell adhesion molecule 5 (IgLON5) antibodies, a distinctive syndrome of sleep disorder, cognitive decline and bulbar onset at the junction of autoimmunity and neurodegeneration [18^▪▪^], and in PNS with anti-Hu antibodies, have also been refined [19^▪^]. Discussion will also extend to publications on epigenetics, including studies of microbiome and somatic tumour mutations.

## BEYOND THE HUMAN LEUCOCYTE ANTIGEN: NEW RISK LOCI IN AUTOIMMUNE ENCEPHALITIS

The first AE GWAS with discovery and validation cohorts involved LGI1-Ab-E. This study revealed two new risk loci which were delineated in 131 French LGI1-Ab-E compared to UK Biobank controls, and recapitulated in 126 LGI1-Ab-E from the United Kingdom, the USA, and Ireland [16^▪▪^].

The strongest finding was a hit of an intronic single nucleotide polymorphism (SNP) rs445608 in *PTPRD*, a protein tyrosine phosphatase with dual immune and brain activity, attaining genome-wide significance in both cohorts [16^▪▪^]. The PTPRD protein is richly expressed in the central nervous system (CNS) [20], has a role in synaptic pruning [21], and like LGI1 itself is downregulated in glioma [22,23]. *In silico* models propose networked interactions with LGI1 and related entities [16^▪▪^]. It has been implicated in several other neurological conditions including restless legs syndrome [24], epilepsy [25], and MS progression [25], as well as involved in clearance of pathogenic tau species [26].

**FIGURE 2:**
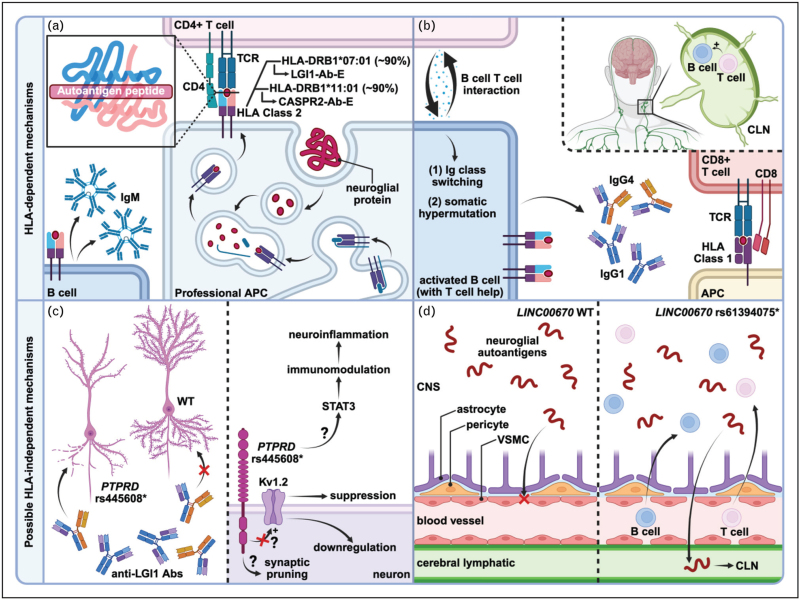
Possible genetic mechanisms in autoimmune encephalitis. (a) The presentation of autoantigenic peptides by professional antigen-presenting cells (APCs) involves a breach in immune tolerance and possession of an appropriate human leukocyte antigen (HLA) allele. Antigenic peptides undergo endocytosis, processing and loading onto HLA class 2 molecules. If host T cell receptors (TCRs) also recognise the presented peptides, T cells can activate B cells leading to generation of autoreactive antibodies. (b) B cell T cell interaction initiates immunoglobulin (Ig) class switching and somatic hypermutation, facilitating the production of the higher affinity immunoglobulin G (IgG). The process of autoimmunisation is currently understood to occur in cervical lymph nodes (CLNs), at least in part. Furthermore, HLA class 1 molecules of the appropriate allele, if again accompanied by the presence of appropriate TCRs, may similarly activate a class of autoreactive cytotoxic T cells. (c) Two significant risk loci have recently been identified through genome-wide association studies (GWAS) of leucine-rich glioma-inactivated protein 1-antibody encephalitis (LGI1-Ab-E), both within intronic regions. The first, involving protein tyrosine phosphatase receptor type delta (*PTPRD*), is implicated in synaptic pruning, which could facilitate synaptic binding of LGI1 autoantibodies. Other possible mechanisms include downstream effects on neuronal excitability via potassium voltage-gated channel subfamily A member 2 (Kv1.2) and immunomodulation via the signal transducer and activator of transcription 3 (STAT3) pathway. (d) The second, involving long intergenic nonprotein coding RNA 670 (*LINC00670*), is upstream of a smooth muscle gene. Speculated mechanisms involve its effects on blood-brain barrier permeability, facilitating the ingress of inflammatory cells into the central nervous system (CNS). Figure created with BioRender. Abs, antibodies; APC, antigen-presenting cell; CASPR2-Ab-E, contactin-associated protein-like 2-antibody encephalitis; CD4, cluster of differentiation 4; CD8, cluster of differentiation 8; CLN, cervical lymph node; CNS, central nervous system; HLA, human leukocyte antigen; Ig, immunoglobulin; IgG1, immunoglobulin G1; IgG4, immunoglobulin G4; IgM, immunoglobulin M; Kv1.2, potassium voltage-gated channel subfamily A member 2; LGI1, leucine-rich glioma-inactivated protein 1; LGI1-Ab-E, LGI1-antibody encephalitis; LINC00670, long intergenic nonprotein coding RNA 670; PTPRD, protein tyrosine phosphatase receptor type delta; STAT3, signal transducer and activator of transcription 3; TCR, T cell receptor; VSMC, vascular smooth muscle cell; WT, wild type. ^*^The single nucleotide polymorphisms discussed have yet to be demonstrated as causative mechanisms in any autoimmune encephalitides, but have been recently identified as genome-wide significant risk loci.

Recently, phospho-tyrosine kinase inhibition was suggested as a physiological response to KV_1.2_ channel excitability secondary to LGI1 insufficiency, owing to antibodies or other factors, proposing a specific mechanistic pathway for this finding [27]. An alternative or even complementary process is suggested by the known epistatic interaction between class I HLA alleles and ERAP1 in multiple autoimmune diseases including ankylosing spondylitis and psoriasis, whereby ERAP-mediated peptide trimming primes allele-restricted antigen presentation [28,29]. By analogy, PTPRD-dependent synaptic pruning could predispose synaptic binding of LGI1-antibodies as part of a pathophysiological cascade of a multihit disease model. (Figs. 1 and 2). Furthermore, PTPRD has been suggested as a druggable target not only for neurological conditions but in metabolic syndrome and obesity, where it plays an important role as a receptor for the appetite-arousing hormone asprosin [30].

A second genome-wide significant risk locus mapped to *LINC00670*, which was also highlighted in a 2022 GWAS of neurological autoimmunity associated with glutamic acid decarboxylase (GAD) antibodies [31]. This long noncoding (lnc) RNA is upstream of a smooth muscle gene *MYOCD*. Lnc RNAs have diverse regulatory roles in close and distant genomic neighbourhoods [32]. It is possible *LINC00670* plays a part in an immune or neurological pathophysiological cascade relevant to AE susceptibility at a class level (Fig. 2), either via this mechanism or an effect on smooth muscle [33] and blood-brain barrier permeability.

**FIGURE 3:**
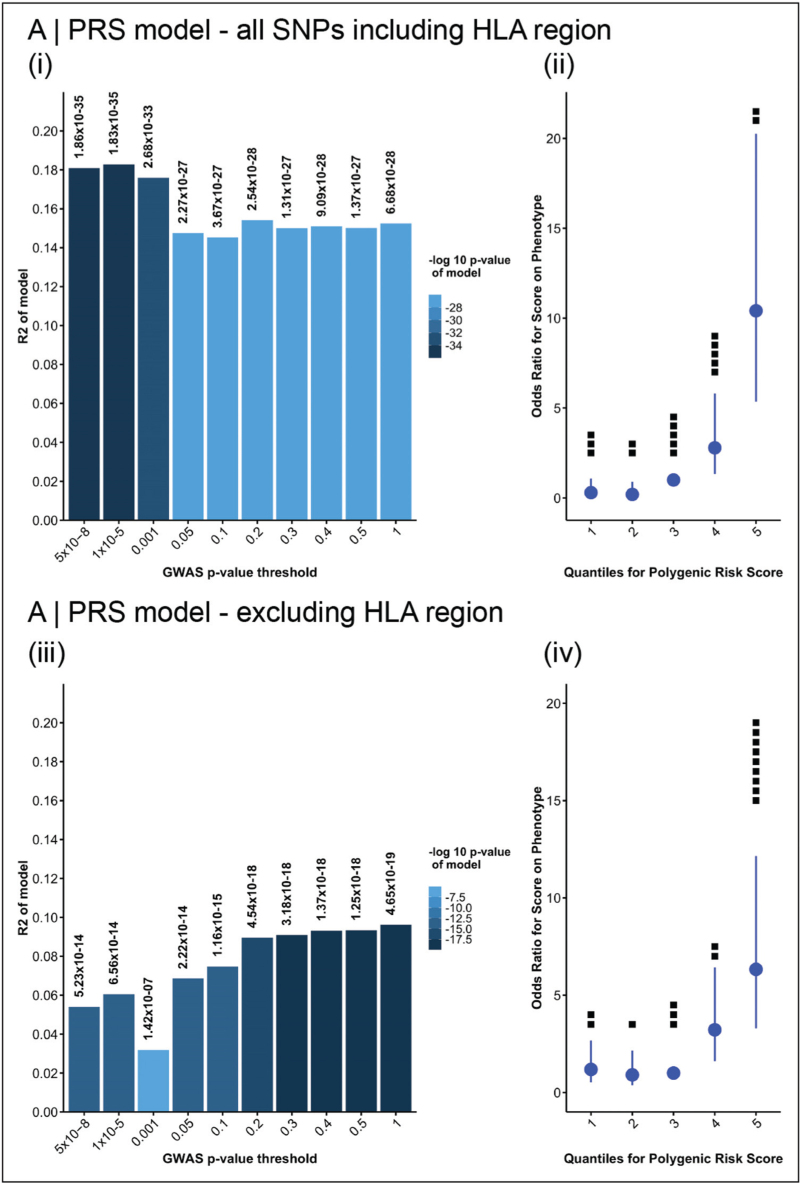
Polygenic risk scores (PRS) in LGI1-antibody encephalitis. (a(i–iv)) Scores calculated passing in all SNPs in chromosomes 1–22 to the bioinformatics tool PRSice. (a(i)) PRS at different levels of GWAS significance (*x* axis) and the proportion of the phenotype accounted for by the model (*y* axis). The significance of each model is shown the *P*-value on top of each bar. (a(ii)) odds ratio of developing the phenotype at each of five quantiles of the PRS. Black dots show numbers of DRB1*07:01 negative patients in each risk quantile. (a(iii–iv)) show the same plots for the PRS calculated excluding the HLA region on chromosome 6 (chr6:25607979–33607978). Note that the number of DRB1*07:01 negative patients is enriched in the top risk quantile when the HLA region is removed. Reproduced with adaptation from Binks *et al.* [16^▪▪^]. This is an open access article distributed under the terms of the Creative Commons CC BY license, which permits unrestricted use, distribution, and reproduction in any medium, provided the original work is properly cited. GWAS, genome-wide association study; HLA, human leucocyte antigen; PRS, polygenic risk score.

Four additional hits were identified by meta-analysis, and a polygenic risk score (PRS) could account for a significant proportion of disease risk, both with and without HLA variants (Fig. 3). This hints at a complex disease architecture with other mediators yet to be delineated.

Somatic mutations in *PTPRD* have been implicated in marginal cell lymphoma [34]. Accordingly, a 2024 targeted sequencing study of 52 Korean LGI1-Ab-E patients examined a panel of genes implicated in clonal haematopoiesis of indeterminate potential (CHIP) [35^▪^]. This is an age-related process whereby bone marrow stem cells acquire mutations potentially predisposing to autoimmune disease or malignancy. Three carried truncating mutations in *ASXL1*, an acute myeloid leukaemia risk gene [35^▪^]. This was enriched compared to ethnically-matched controls, but the small sample size calls for replication in larger datasets [35^▪^].

Prior to the time under consideration in this summary, there were two GWAS of N-methyl D-aspartate-receptor encephalitis (NMDAR-Ab-E). The first, in 2018, which also studied 54 LGI1-Ab-E patients, included 96 NMDAR-Ab-E individuals but did not find any signals [36]. This was likely due to a combination of small sample size and population factors. A follow-up GWAS in 2023 included most of the same patients but expanded the total cohort to 178 and was more stringent on the included population [37]. Two risk loci, comprising three potential risk genes, were reported. One, in *LRRK1* (chromosome 15), which has a role in B cell activation [37]; the second, on chromosome 11, with several candidates including the transcription factor *NR1H3 liver X receptor alpha*, and *ACP2*, a lysosomal acid phosphatase 2 recently identified in a high-throughput screen as a potential regulator of type 1 interferon responses [38].

A further GWAS from China in 2024 did not replicate these hits, but did also alight on type 1 interferon response elements [17^▪^]. Its chief hit was the *IFIH1* interferon locus on chromosome 2. *IFIH1* is an established mediator of effective antiviral responses, as well as a risk factor in numerous autoimmune conditions [39]. These include ankylosing spondylitis, Crohn's disease, type 1 diabetes, psoriasis, systemic lupus erythematosus, and the rare genetic CNS inflammatory syndrome Aicardi-Goutières [40]. The lead SNP, rs3747517, is likely to be of functional relevance since it is a mis-sense variant situated in an exon and associated with expression levels of IFIH1 in the anterior cingulate cortex and cerebellar cortex [17^▪^]. This GWAS also confirmed a previous weak association with HLA-DRB1*16:02 in Chinese patients, and proposed a further risk HLA, HLA-DQB1*05:02, albeit limited to 15% of patients (Table 1).

Overall, these findings are beginning to decipher non-HLA contributors to AE immunogenetics. These extra-HLA loci are involved in both innate and adaptive immunity and probably combine with existing susceptibility provided by carrying a risk HLA to increase genetic risk. Nevertheless, overall extant GWAS are small, in part an inevitable consequence of rare disease, but large multiethnic collaborations will likely be mandatory, as recently published in narcolepsy [41^▪▪^], to power further discoveries.

## REFINING HUMAN LEUCOCYTE ANTIGEN ASSOCIATIONS IN AUTOIMMUNE ENCEPHALITIS AND PARANEOPLASTIC NEUROLOGICAL SYNDROMES

Despite these developments, HLA remains the dominant immunogenetic risk factor across most AE and some PNS forms, and is most integrated in the clinical sphere (Table 1). For example, in LGI1-antibody encephalitis, the most common adult AE, marked by frequent seizures, personality change and cognitive decline, the risk of disease is 27-fold in DRB1*07:01 carriers [12]. HLA typing can form part of diagnostic work-up, refining prior probabilities, particularly in subtle or ambiguous presentations. However, it was previously revealed that an association with a rare allele, DRB1*04:02, is also found in some people with LGI1-antibodies, particularly younger patients and women [14].

In 2025, a study of 228 French, Italian and Spanish patients, 175 with HLA information available, delved into the clinical association of these HLAs in more detail [42^▪▪^]. The study confirmed the previous literature of a younger (median age 59 vs. 67, *P* = 0.005) and nonpredominantly male (9/19, 47% vs. 107/156,69%; *P* = 0.044) profile of DRB1*07:01 noncarriers. It also appended some potentially important disease-relevant aspects, because noncarriers of either the 07:01 or 04:02 allele were more likely to have an associated cancer, including thymoma, and worse disease trajectory [42^▪▪^].

This is relevant to management, suggesting that patients without a recognized risk HLA might be considered for a more extensive cancer work-up and require assertive therapy. It can also be a factor in counselling to patients and family on likely disease outcomes. The authors postulated that these poorer outcomes could be underpinned by the propensity of HLA-associated diseases to produce antibodies of the immunoglobulin G4 (IgG4) sub-class [42^▪▪^]. These act predominantly via receptor blockade, by contrast to the known ability of IgG1 sub-class antibodies to trigger complement and cause long-lasting tissue damage.

Another HLA association which was refined recently concerned IgLON5-antibody associated disease. This entity, first described in 2015, can be thought of as an overlap disorder spanning both autoimmune neurology and neurodegeneration [43]. Distinctive features include a non-REM sleep behaviour disorder and bulbar symptoms, with accompanying encephalopathy rather than encephalitis transitioning to tau deposition and neurodegeneration if unchecked. From the first, a strong class 2 HLA association was described, and associations with both HLA-DRB1*10:01 (~50%) and HLA-DQB1*05:01 (~75%) were noted [43]. Higher antibody titres were reported with DRB1*10:01, while the prevalence of the characteristic sleep disorder was elevated with either HLA [44].

Subsequently, DQB1*05:01 emerged as the predominant pathogenic mediator, through computational and *in vitro* binding assays [18^▪▪^]. Although several investigators have employed *in silico* methods to identify strong-binding peptides to 07:01 and 04:02 in LGI1-Ab-E [10,12,14,45], the functional depiction of HLA mechanisms stands out in the field to date. Among the points of interest were that the immunogenic peptides were shown to be posttranslationally modified, and the description of CD4^+^ T cell epitopes, pointing to CD4^+^ T and B cell interactions as a pivotal disease stage. Furthermore, T cell epitopes were shown to closely overlap with those previously delineated of B cells, suggestive of a pathomechanistic role of epitope spreading. Additional clinical accompaniments included the finding that, akin to DRB1*07:01 in LGI1-Ab-E, DQB1*05:01 links to male sex, and homozygosity confers a younger age of onset [18^▪▪^].

Another avenue to explore causative epitopes may be a cross-species approach, incorporating the observation that pet cats are known to develop a spontaneous form of LGI1-antibody encephalitis [46–48]. It is not yet known whether these cats also display a ‘HLA’ (known in cats as feline leucocyte antigen) predilection. However, shared leucocyte antigen epitopes have been delineated in dogs and humans prone to another autoimmune condition, rheumatoid arthritis [49].

A new insight into anti-Hu paraneoplastic syndromes was offered in 2024, which highlighted that different haplotypes may underlie different phenotypes found in connection with these antibodies [19^▪^]. The same investigators previously outlined a similar scenario when considering the role of DRB1*11:01 in CASPR2-antibodies, a condition which can have both central and/or peripheral manifestations, finding that this Class 2 was enriched in CASPR2-antibody AE but not in Morvan's syndrome or peripheral nerve phenotypes [13].

While anti-Hu antibodies consistently associate with small-cell lung cancer, like in CASPR2, patients can present with limbic encephalitis, peripheral neuropathy or both central and peripheral features [6]. The recent publication found a DQA1*05:01~DQB1*02:01 ~ DRB1*03:01 haplotype distinguished those with peripheral syndromes, particularly sensory neuropathy but was absent in patients with a central presentation, who carried the DRB4*01:01 allele. Although numbers were small, a tentative connection between DQ2 and DR3 and the presence of a confirmed cancer was also drawn [19^▪^].

These different HLA associations might portend separate disease pathways, which could potentially be impactful in considering therapeutic targets or preferential treatment modalities. One interesting possibility is that the varying HLA alleles could exert influence in the possibility of pathogenic autoantibodies breaching the blood brain barrier. This has been shown in MS, where certain HLAs are linked to the IgG index as well as antibody isotype [50,51]. Class switching is also likely to be an effector mechanism of HLA-mediated susceptibility, at-risk individuals progressing from generating short-lived low-affinity IgM antibodies to IgG entities capable of prolonged tissue attack [52].

## EPIGENETICS AND SOMATIC MUTATIONS

The gut-brain axis is increasingly implicated in the pathogenesis of several neurological diseases including Alzheimer's dementia and Parkinson's disease. Microbiota can induce epigenetic modifications relating to transcription and translation of the host genome and are attractive therapeutic targets due to the potential reversibility of these actions [53]. A study published in 2025 examined the microbiome in 42 patients with LGI1-antibodies and 27 familial or household controls, and found evidence for a shift in composition towards the *Bacteroidetes* phyla potentially associated with HLA dosage, and a composition favouring a loss of anti-inflammatory short chain fatty acids [54^▪^]. The modest size combined with the chronic timepoint of sampling of most participants limits this study's conclusions. However, similar changes had been found in an earlier, smaller study in a Chinese population [55], whereas a reduction in *Bacteroidetes* was observed in 10 Chinese NMDAR-Ab-E [56] patients. It is to be hoped future studies with larger cohorts sampled at disease onset can build on and clarify this information. Relevant considerations could include differing regional dietary habits, and the older age of LGI1- compared to NMDAR-antibody patients.

Somatic genetic variants are known to be relevant in certain paraneoplastic syndromes. Tumours in ovarian cancer-associated Yo cerebellar degeneration are notable for carrying such variants in the Yo antigen, most frequently mis-sense variants, compared to ovarian cancers from women without a paraneoplastic attack [57]. Recently, a similar study was made of ovarian teratomas in women with NMDAR-Ab-E. By contrast, these germline tumours did not yield somatic variants different to nonencephalitic teratoma controls [58^▪^]. Although, as in Yo disease, the tumours serve as foci of immune activity [59], the driver – formation of germinal-like centres – is likely not linked to intrinsic tumour mutations.

## CONCLUSION

Several new developments in the past 18 months highlight an important genetic role in both AE and PNS. Discoveries may already have a purpose as a biomarker, and could in the future guide rational drug development target. The new LGI1 risk locus of *PTPRD* has the potential to unite CNS and immune mechanisms. However, the continued paucity of familial cases [60,61], the frequency of risk HLA alleles in healthy individuals, and the applicability of PRS, point to a multihit model combining genetic and environmental hazards.

## Acknowledgements

*None.*

### Financial support and sponsorship

*Dr Binks is funded by a NIHR Clinical Lectureship. Prof Knight receives support from the Medical Research Council (MR/V002503/1), Chinese Academy of Medical Sciences (CAMS) Innovation 537 Fund for Medical Science [2018-I2M-2-002], and the NIHR Oxford Biomedical Research Centre.*

### Conflicts of interest

*There are no conflicts of interest.*

## REFERENCES AND RECOMMENDED READING

Papers of particular interest, published within the annual period of review, have been highlighted as:

▪ of special interest
▪▪ of outstanding interest
